# Wafer Handing Robotic Arm Vibration Trajectory Planning Based on Graylag Goose Optimization

**DOI:** 10.3390/s26030829

**Published:** 2026-01-27

**Authors:** Yujie Ji, Peiyan Hu

**Affiliations:** School of Mechanical Engineering, Shenyang Ligong University, Shenyang 110159, China

**Keywords:** wafer handing robotic arm, trajectory planning, Graylag Goose Optimization

## Abstract

In contemporary semiconductor manufacturing, wafer-handling robots are essential for achieving high-speed and high-precision wafer transportation. However, the demand for rapid motion and lightweight design introduces flexible transmission components that are prone to residual vibrations, which degrade positioning accuracy and system stability. To address this challenge, this paper proposes a vibration-suppression trajectory planning method based on the Gray Goose Optimization (GGO) algorithm. The proposed algorithm integrates grouped global search with local optimization capabilities, making it well suited for solving multi-objective optimization problems. Comparative tests conducted on eight randomly selected multimodal benchmark functions from the CEC2013 test suite verify the effectiveness and robustness of the GGO algorithm. Establishing a multi-objective function that considers both motion time and vibration energy enables the GGO algorithm to determine the switching time points of an S-shaped velocity profile, thereby generating smooth trajectories with continuous velocity and acceleration. By varying different initial conditions, the trade-off between motion time and vibration energy is systematically analyzed with respect to angular displacement, initial acceleration, and time-weighting factors. Simulation results indicate that the planned trajectories exhibit negligible displacement variation under zero-mean disturbances. The velocity error remains within 0.1 deg·s^−1^, and the acceleration error is confined within 0.2 deg·s^−2^. Consequently, Pareto-optimal solutions are successfully obtained with respect to both motion time and residual vibration energy.

## 1. Introduction

The wafer transport robotic arm is an important actuator in semiconductor manufacturing equipment, and its performance directly affects production throughput and yield. As semiconductor process nodes continue to shrink, stringent requirements are placed on the robotic arm’s positioning accuracy, stability, and operational efficiency [1,2,3]. Traditional time-optimal trajectory planning methods often focus on achieving the fastest point-to-point motion of the robotic arm’s end effector while neglecting the issue of residual vibrations at the end effector caused by the flexible characteristics of the transmission system. Such vibrations can lead to a decrease in the positioning accuracy of the robotic arm’s end effector, and in severe cases, may cause wafer displacement or breakage, resulting in reduced productivity and significant economic losses [4,5]. Therefore, efficient and precise vibration-suppression trajectory planning has become one of the core technologies in the development of advanced wafer robots.

The vibration suppression methods for robotic arms can be categorized into passive and active vibration suppression. Passive vibration suppression primarily involves optimizing the topology to improve mass distribution, using lightweight, stiff materials to enhance the equivalent stiffness, and incorporating damping devices and energy-absorbing components to suppress vibrations through physical mechanisms. These passive vibration suppression schemes do not require external energy input, are cost-effective, but have limited adaptability to time-varying operating conditions, making them difficult to meet the requirements of wafer transport robotic arms [6]. Active vibration suppression, on the other hand, reduces the vibrations of the robot by improving control strategies, thereby enhancing motion precision and stability. PID control, with its simple and intuitive structure and low implementation difficulty, is widely used in the motion control of industrial robots to suppress elastic vibrations in robotic arms. Yang C et al. employed PID fuzzy control to suppress elastic vibrations in flexible robotic arms, improving the positioning and control accuracy of piezoelectric flexible arm ends [7]. Guo et al. proposed a tracking control system integrating an incremental PID controller and a sliding mode robust controller, which demonstrated stronger accuracy, stability, and anti-interference capabilities compared to traditional PID control [8]. In addressing similar robotic arm control problems, Wazzan utilized PID sliding mode control combined with a genetic algorithm to tune controller parameters, effectively improving the performance of the robotic arm’s PID controller [9]. Despite the simplicity and intuitiveness of these control methods, these controllers are often optimized based on specific models, and their control parameters are difficult to meet the requirements for high precision and stability, especially when dealing with complex robotic arm structures or variable working environments.

Recently, researchers have commonly employed vibration suppression trajectory optimization to address the limitations of both passive and active vibration suppression, providing a more cost-effective and universally applicable solution for robotic arm vibration control. This approach has become one of the key research focuses in the field of robotic arm control [10]. These methods formulate trajectory planning as a multi-objective or constrained optimization problem by simultaneously considering system dynamics, motion time, energy consumption, joint smoothness, and end-effector vibration, and they commonly introduce metaheuristic optimization algorithms to search for optimal solutions. These optimization algorithms, inspired by natural phenomena and biological behaviors, offer new approaches for solving trajectory planning and constrained optimization problems of this nature [11]. The development of metaheuristic optimization algorithms can be broadly divided into two directions: global search and local optimization. Among them, the Genetic Algorithm (GA) is widely used in various global search optimization problems due to its strong capability in handling complex constraints, combinatorial optimization, and global exploration. Xu Qiang et al. proposed a genetic algorithm combined with a simulated annealing mechanism, which further improved the optimization accuracy, shortened the robotic arm’s motion time, and enhanced its work efficiency [12]. Meanwhile, Kheshti et al. applied genetic algorithms to adjust the gain optimization of sliding mode controllers, reducing the time required for a manipulator arm to complete a predefined motion trajectory [13]. However, the computational model of genetic algorithms is relatively complex, and its use has certain limitations. In contrast, Particle Swarm Optimization (PSO) has a simpler algorithm structure and stronger local optimization capability. In this context, Wu N et al. proposed an improved particle swarm optimization algorithm (RTPPSO) to optimize the robotic arm’s joint angles or path. By introducing an adaptive weight strategy, they significantly improved the robotic arm’s work efficiency [14]. However, when solving complex problems with multiple dimensions, strong constraints, and strong coupling, PSO is prone to accuracy degradation and insufficient global search capability due to the influence of random disturbances [15,16,17]. Researchers have proposed various new optimization algorithms and introduced different solution strategies to address the shortcomings of these two algorithms. Yue et al. applied a gray wolf optimization algorithm with a probabilistic disturbance strategy to solve the time-optimal problem. This optimization strategy effectively balanced the weight between local optimization and global search [18]. However, the gray wolf population may converge around suboptimal individuals, leading to premature convergence issues [19,20]. Pu Q et al. introduced an improved wild dog optimization algorithm for robotic arm time-impact optimization [21,22], but they did not perform tests for solving high-dimensional problems using the wild dog optimization algorithm. Additionally, to improve global search capability and address the issue of search direction disturbance, Lv Yun peng et al. proposed an inverse kinematics solver based on a multi-strategy improved dung beetle optimization algorithm. However, this improved algorithm has not been applied to the multi-objective vibration optimization direction [23]. Widyianto Agus et al. addressed the issue of the shipboard two-degree-of-freedom robotic arm being affected by environmental factors and proposed a real-time tuning of PD control parameters based on an ant colony algorithm, significantly improving the stability of shipboard equipment [24]. Abro et al. combined ant colony optimization’s pheromone learning with the Denavit–Hartenberg (DH) method to develop a robotic arm error model, further enhancing the accuracy of global search and improving the reliability and accuracy of industrial robotic systems [25]. However, the ant colony algorithm is heavily dependent on the initial model, making it difficult to avoid local optimal solutions. The Whale Optimization Algorithm (WOA) introduced a random spiral search step, eliminating the need for crossover and mutation operators, with a simpler program structure. Wang Fang et al. applied a hybrid whale optimization algorithm to optimize the time-impact optimal trajectory of industrial robotic arms, using fifth-order B-splines for interpolation planning of motion trajectories in joint space [26]. However, the performance of this algorithm significantly deteriorates in a multi-constraint environment and requires further integration with various improvement strategies [27]. The Graylag Goose Optimization (GGO) algorithm introduced a group migration strategy [28], demonstrating strong search capabilities in multi-objective problems and efficient local optimization when facing suboptimal solutions. Ashish Sharma et al. conducted eight multi-objective benchmark tests, with the results showing that the Multi-Objective Graylag Goose Optimization (MOGGO) algorithm exhibited excellent performance in terms of convergence [29].

In summary, this paper introduces the GGO algorithm into the vibration suppression trajectory optimization problem of wafer transport robotic arms, providing a new feasible solution for multi-objective trajectory optimization. This study constructs the end dynamics equation of the flexible lower arm and integrates a multi-objective optimization model involving motion time, energy, and impact. Based on the multi-objective GGO algorithm, the trajectory parameters are solved, aiming to generate a set of Pareto-optimal trajectories with superior overall performance, offering a novel and efficient solution to improve the performance of the wafer transport system.

## 2. Establishment of the Dynamics Model for Wafer Transport Robotic Arm

**Figure 1:**
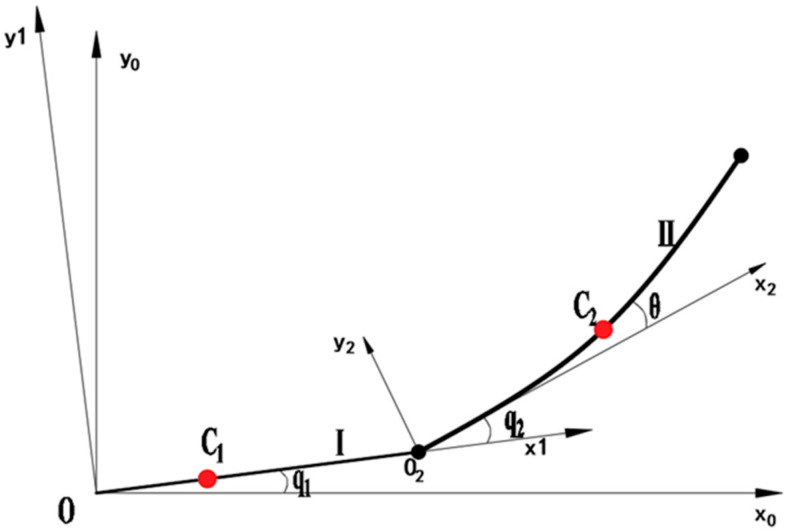
Motion Model of Wafer Transport Robotic Arm.

In this study, the wafer transport robotic arm mainly consists of two links [30,31]. The upper (first) link is rigidly connected to the motor, while the lower (second) link is driven by another motor via a flexible steel belt inside the upper link, which can be regarded as a flexible motion joint. During motion, only the bending stress of the steel belt is considered. The end-effector is coupled to the posture of the upper link through the steel belt embedded inside the lower link. As shown in Figure 1, *q*1 and *q*2 denote the rotational joint angles defined by the DH parameters, whereas *θ* represents the elastic deformation angle of the flexible transmission element.

Table 1 shows the simplified model of the wafer transport robot, which consists of the rigid link I (upper arm) and the flexible link II (lower arm). The world coordinate system X_0_OY_0_ is established with the base as the origin, the coordinate system X_1_OY_1_ is set at the end-point of link I (upper arm), and the coordinate system X_2_O_2_Y_2_ is established at the end-point of link II (lower arm). C_1_ and C_2_ represent the center of mass of the two links. *q*_1_ is the rotation angle of the primary arm, *q*_2_ is the rotation angle of the secondary arm, and *θ* is the deformation of the flexible lower arm. The structural parameters of the wafer transfer robotic arm in this study are shown in Table 2.

First, based on the Lagrange method, the dynamic equations of a planar two-degree-of-freedom rigid robot are established. First, the robot’s potential energy equation is analyzed. The robot’s forearm can be regarded as a flexible arm rod, and its elastic potential energy can be expressed as

(1) $$V_{e}=\frac{1}{2}k(q^{2}-\theta^{2})$$

Here, the *θ* refers to the ideal angle of the forearm when no deformation occurs. Since the robot prototype moves parallel to the ground throughout, gravitational potential energy is not taken into account. The kinetic energy expression includes both the rotational kinetic energy of each link and the translational kinetic energy of the link centers of mass.

Then the total kinetic energy of the robotic arm is:

(2) $$T=T_{1}+T_{2}=\frac{1}{2}m_{1}v_{c1}^{2}+\frac{1}{2}I_{1}{\dot{q}}_{1}^{2}+\frac{1}{2}m_{2}v_{c2}^{2}+\frac{1}{2}I_{2}{\left({\dot{q}}_{1}+{\dot{q}}_{2}\right)}^{2}$$

I_1_ and I_2_ represent the moments of inertia of the link about its center of mass. Since both the upper arm and forearm rotate around their joints, the moments of inertia of the upper arm and forearm are:

(3) $$I_{1}=\frac{1}{3}m_{1}l_{1}^{2},I_{2}=\frac{1}{3}m_{2}l_{2}^{2}$$

The Lagrange function of the robotic arm is:

(4) $$L=T-V=\frac{1}{2}(I_{1}+m_{2}l_{1}^{2}+I_{2}){\dot{\theta }}_{1}^{2}+\frac{1}{2}I_{2}{\dot{\theta }}_{2}^{2}+I_{2}{\dot{\theta }}_{1}{\dot{\theta }}_{2}+m_{2}l_{1}l_{c2}{\dot{\theta }}_{1}({\dot{\theta }}_{1}+{\dot{\theta }}_{2})\cos\theta_{2}-\frac{1}{2}k{\left(\theta_{2}-\theta_{o}\right)}^{2}$$

Based on the Lagrange function, the Lagrange equations are further applied to each generalized coordinate.

The Lagrange dynamic equation of the lower arm is:

(5) $$\frac{d}{dt}\left(\frac{\partial L}{\partial {\dot{q}}_{1}}\right)-\frac{\partial L}{\partial q_{1}}=\tau_{1}$$

The Lagrange dynamic equation of the upper arm is:

(6) $$\frac{d}{dt}\left(\frac{\partial L}{\partial {\dot{q}}_{2}}\right)-\frac{\partial L}{\partial q_{2}}=\tau_{2}$$

The dynamic model of the wafer transfer robot system is:

(7) $$\left[\begin{array}{c} \tau_{1}\\ \tau_{2} \end{array}\right]=\left[\begin{array}{cc} M_{11} & M_{12}\\ M_{21} & M_{22} \end{array}\right]\left[\begin{array}{c} {\ddot{q}}_{1}\\ {\ddot{q}}_{2} \end{array}\right]+\left[\begin{array}{cc} C_{11} & C_{12}\\ C_{12} & C_{22} \end{array}\right]\left[\begin{array}{c} {\dot{q}}_{1}\\ {\dot{q}}_{2} \end{array}\right]+\left[\begin{array}{c} 0\\ k(q_{2}-\theta ) \end{array}\right]$$

In Equation (7), the stiffness term *k*(*q*_2_ − *θ*) represents the restoring torque induced by the elastic deformation of the flexible transmission and is applied to the corresponding joint equation.

## 3. Vibration Suppression Trajectory Planning for Robotic Arms Based on Graylag Goose Algorithm

**Figure 2:**
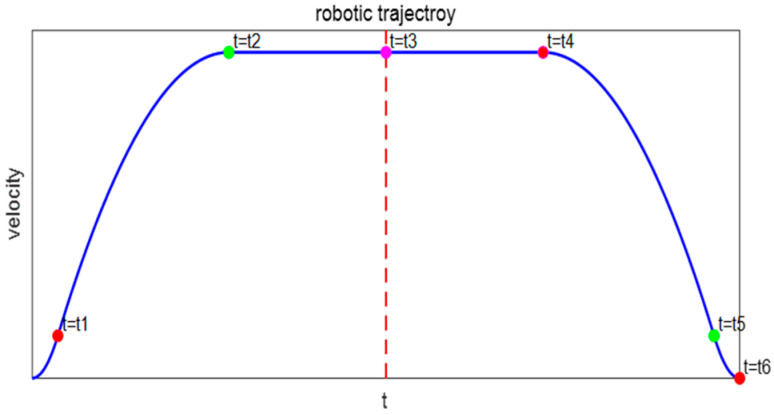
Velocity planning S-shaped curve.

Existing robot trajectory planning methods include cubic and quintic polynomial trajectory planning algorithms [32,33,34], T-shaped and S-shaped acceleration-deceleration trajectory planning algorithms, and B-spline trajectory planning algorithms. During the operation of a wafer handling manipulator, the start and end positions are known, while the motion states during the process are unknown. This study uses a cubic polynomial interpolation method to plan the motion trajectory [35]. This method combines the low impact and high stability of S-shaped trajectory planning with the low computational load of T-curve planning. It is easy to program and debug, has high reliability, and can avoid sudden changes in acceleration, thereby effectively suppressing vibrations at the manipulator’s end. As shown in Figure 2, the polynomial trapezoidal interpolation curve trajectory is defined over the interval (0, t_6_). The robotic arm first experiences smooth startup acceleration, the acceleration decreases beginning at time t_1_, and reaches constant velocity at time t_2_. If t = t_4_, the speed begins to decelerate until it reaches 0 at t_6_, achieving a smooth stop. The trajectory expression is given in Equation (8).

(8) $$\dot{q}=\left\{\begin{array}{c} \begin{array}{c} at^{2},0<t<t_{1}\\ bt^{2}+ct+d,t_{1}<t<t_{2} \end{array}\\ \begin{array}{c} bt_{2}^{2}+ct_{2}+d,t_{2}<t<t_{4}\\ b^{\prime }t^{2}+c^{\prime }t+d^{\prime },t_{4}<t<t_{5}\\ a{\left(t-2t_{3}\right)}^{2},t_{5}<t<t_{6} \end{array} \end{array}\right.$$

Let the angular displacement of the wafer transport robotic arm be A, then its displacement equation can be expressed in Equation (9).

(9) $$\frac{2}{3}at_{1}^{3}+\frac{2}{3}bt_{2}^{3}+ct_{2}^{2}+2dt_{2}-\frac{2}{3}bt_{1}^{3}-ct_{1}^{2}-2dt_{1}+2(t_{3}-t_{2})(bt_{2}^{2}+ct_{2}+d)=A$$

(10) $$P=\frac{1}{3}at_{1}^{3}+\frac{1}{3}bt_{2}^{3}+\frac{1}{2}ct_{2}^{2}+dt_{2}-\frac{1}{3}bt_{1}^{3}-\frac{1}{2}ct_{1}^{2}-dt_{1}$$

(11) $$Q=bt_{2}^{2}+ct_{2}+d$$

For the convenience of deriving the formula, we replace part of the polynomial in the displacement formula with P and Q, as shown in Equations (10) and (11).

The rate of change in jerk directly affects the efficiency of these energy losses, especially during the initial impact or rapid change stages. In a vibration system, the integral over time of the square of the jerk reflects the residual vibration energy of the system during rapid start-stop processes. To quantitatively evaluate vibration suppression performance, Equation (12) defines the residual vibration energy as the time integral of the squared jerk. Since jerk represents the rate of change in acceleration, minimizing this index effectively suppresses high-frequency excitation and residual oscillations during rapid start–stop motions.

In this study, the planned velocity profile is designed to be symmetric with respect to the mid-time instant t_3_. As a result, the jerk-related vibration energy contributed by the second half of the motion [t_3_, t_6_] is identical to that of the first half [t_0_, t_3_]. Therefore, for conciseness, Equation (12) evaluates the vibration energy over [t_0_, t_3_], and the total vibration energy over the complete motion interval [t_0_, t_6_] can be obtained.

(12) $$E_{vib}=\int_{0}^{t_{3}}\left(\dddot{q}(t\right){)}^{2}dt$$

The multi-objective optimization function is:

(13) F(t1,t2)=ω1t2+30−PQ+ω24a2t1+4at1t1−t22(t2−t1)Min

Optimization is the process of finding the best solution to a problem among all possible alternatives [36,37,38]. The Graylag Goose Optimization (GGO) algorithm, as an emerging metaheuristic algorithm, is inspired by the intelligent behaviors of gray geese during long-distance migration, such as efficient formation flying, cooperative resting, and dynamic leadership. The algorithm simulates mechanisms like “leader goose switching,” “flock following,” and “energy conservation,” enabling a finer balance between convergence accuracy and optimization speed.

The Graylag Goose Optimization (GGO) algorithm consists of two stages: global exploration and local optimization. Based on the constraints of the objective function, a fitness function is defined. After setting the population size, all randomly generated individuals enter the initial exploration group. The population is then sorted according to fitness values, and the top N solutions in terms of fitness are defined as the exploitation group, while the remaining poorer solutions form the exploration group. Individuals that meet the fitness function criteria are assigned to the “exploitation group,” whereas those that do not are given high penalty values and re-enter the exploration group to search for solutions. The initial parameters of the population are shown in Table 3.

First, randomly generate N arbitrary solutions. The individual position update strategy of the Graylag Goose Optimization Algorithm is divided into two parts: global search by the exploration group and local optimization by the exploitation group. If an individual’s value does not meet the requirements of the fitness function, assign a high penalty value to that individual.

Explore group global search:

Strategy 1: Move toward the global best solution X_best_.

(14) $$x_{\mathrm{new}}=x_{best}-A\cdot \left|C\cdot x_{best}-x\right|$$

(15) $$A=2(1-iter/MaxIter)\cdot r_{1}+(1-iter/MaxIter),\;C=2r_{2}$$

The r_1_ and r_2_ is random Vector

Strategy 2: Guided by Three Random Geese.

(16) $$x_{new}=\omega_{1}x_{r1}+z\omega_{2}(x_{r2}-x_{r3})+(1-z)\omega_{3}\left(x-x_{r1}\right)$$

Partial optimization by the development team

Strategy 1: The three-sentinel geese seek the average value, where the sentinel geese individuals are selected from the three best solutions in the group based on fitness.

(17) $$x_{new}=\frac{1}{3}\left(x_{s1}+x_{s2}+x_{s3}\right)$$

Strategy 2: Perturbation Near the Current Optimal Solution.

(18) $$x_{new}=x+0.5(1+z)\omega (x-x_{best})$$

**Figure 3:**
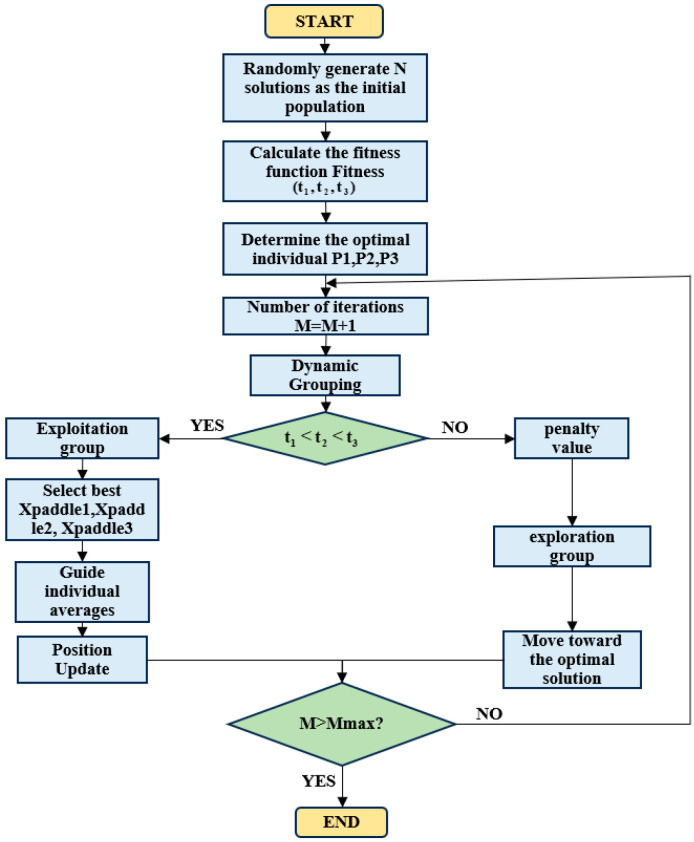
Logical Flowchart of the Graylag Goose Optimization Algorithm.

If the maximum number of iterations is reached, it is considered that the final solution has been achieved, and the iteration stops, exporting the current optimal solution. And its logical sequence diagram is shown in Figure 3.

## 4. Algorithm Testing and Analysis

To verify the performance of the multi-objective GGO function in different workspaces, five metaheuristic optimization algorithms—Particle Swarm Optimization (PSO) [14], Gray Wolf Optimizer (GWO) [18], Whale Optimization Algorithm (WOA) [26], Non-dominated Sorting Genetic Algorithm II (NSGAII) [10], and Dung Beetle Optimization (DBO) [23]—were added on the MATLAB 2022B platform for comparison with the multi-objective GGO function. Two unimodal functions, four multimodal functions, and two composite functions from the CEC2013 benchmark test set were selected as objective functions, as shown in Table 4. The performance of the multi-objective GGO algorithm was evaluated by comparing the results of the test set runs.

In Table 4, a subset of benchmark functions from the CEC2013 test suite is selected to evaluate the optimization performance of the proposed algorithm. Specifically, functions F4 and F5 are representative unimodal functions used to assess exploitation capability and convergence accuracy, whereas F9, F14, F15, F16, F21, and F23 are multimodal or composite functions designed to evaluate global search ability and robustness against local optima.

These functions cover different landscape characteristics, including high dimensionality, strong nonlinearity, and multiple local minima, which are consistent with the complexity of multi-objective trajectory optimization problems in robotic systems.

Based on the original Gray Goose Optimization (GGO) algorithm, this study further develops an improved multi-objective variant to better address the dual-objective vibration suppression trajectory optimization problem.

**Figure 4:**
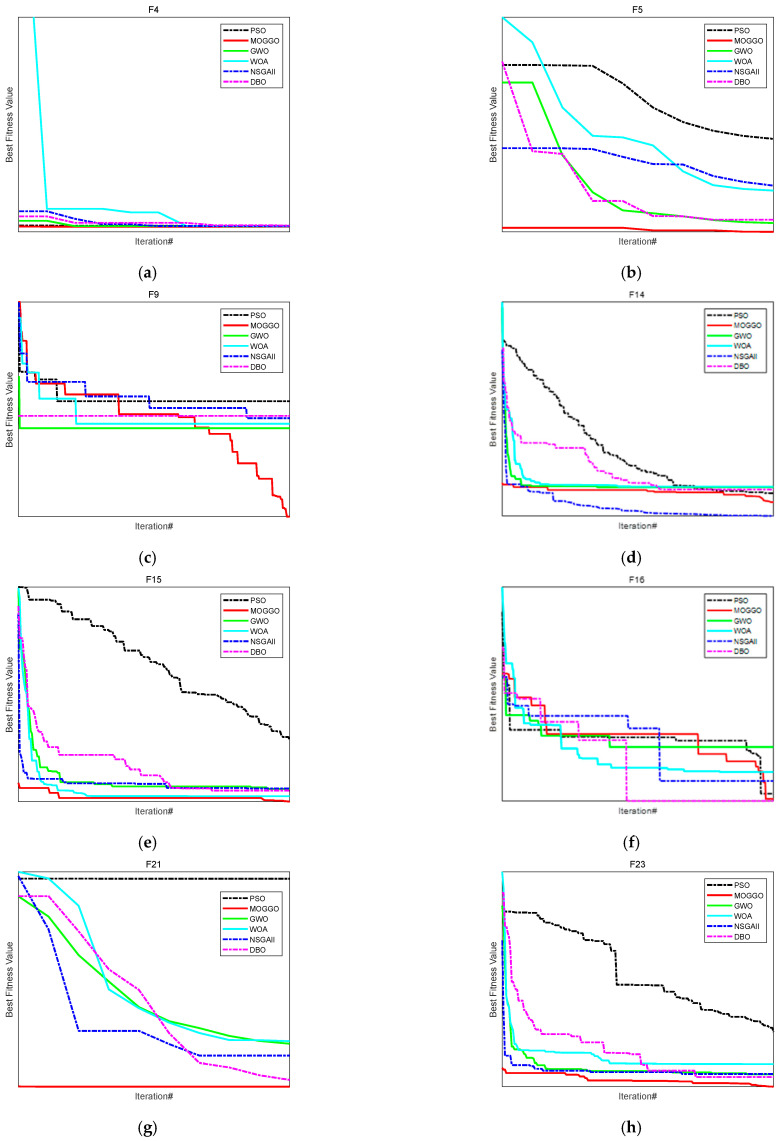
Convergence Curves for a Subset of the CEC2013 Test Functions.

It should be noted that the proposed Multi-Objective Gray Goose Optimization (MOGGO) algorithm is an improved version of the original GGO algorithm. In this study, a Logistic chaotic map is introduced to enhance the diversity of the initial population, while a Gaussian (normal) disturbance mechanism is incorporated during the optimization process to improve global exploration ability and avoid premature convergence. In addition, the original single-objective GGO framework is extended to handle multi-objective optimization, making it suitable for solving the dual-objective vibration suppression trajectory planning problem. The results of the CEC2013 test set are shown in Figure 4. The results indicate that MOGGO performs the best overall on the CEC2013 test functions, significantly outperforming the comparison algorithms in terms of convergence speed, solution accuracy, and stability.

As shown in Figure 4a–h, the convergence behaviors of different algorithms are compared on benchmark functions F4, F5, F9, F14, F15, F16, F21, and F23. Overall, MOGGO demonstrates faster convergence speed and better solution accuracy across most test functions. In unimodal functions, the MOGGO rapidly reaches the optimal region, while in multimodal and complex functions, it maintains stable convergence and effectively avoids premature stagnation. In contrast, PSO, WOA, GWO, and NSGA-II generally exhibit slower convergence or are more prone to local optima. These results indicate that MOGGO achieves a better balance between global exploration and local exploitation.

(1) Set the forearm to rotate 120°, with the motor’s starting acceleration at 20 deg/s^2^. On the MATLAB platform, set the population size and number of iterations, define the main loop function of the algorithm and the displacement equation, and establish it as a fitness function according to the strict constraint t_1_ < t_2_ < t_3_. The fitness function can be expressed as:

(19) $$F=\omega_{1}t_{3}+\omega_{2}E_{vib}+\varepsilon$$

Among them, _1_ and _2_ are the target weight values, and e is the penalty value. In Equation (19), *w*1 and *w*2 denote the weighting coefficients for motion time and residual vibration energy, respectively, satisfying *w*_1_ + *w*_2_ = 1. These parameters allow flexible trade-offs between efficiency and vibration suppression according to different operational requirements.

It is triggered when an individual does not satisfy the condition t_1_ < t_2_ < t_3_, and is randomly assigned a larger value to make the individual enter the exploration group to perform a deep search.

**Figure 5:**
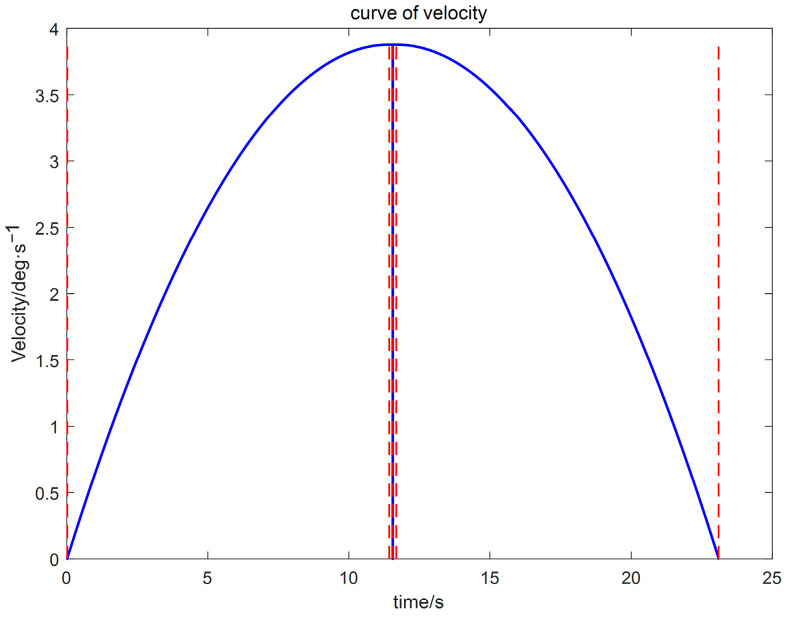
Velocity trajectory planning profile.

**Figure 6:**
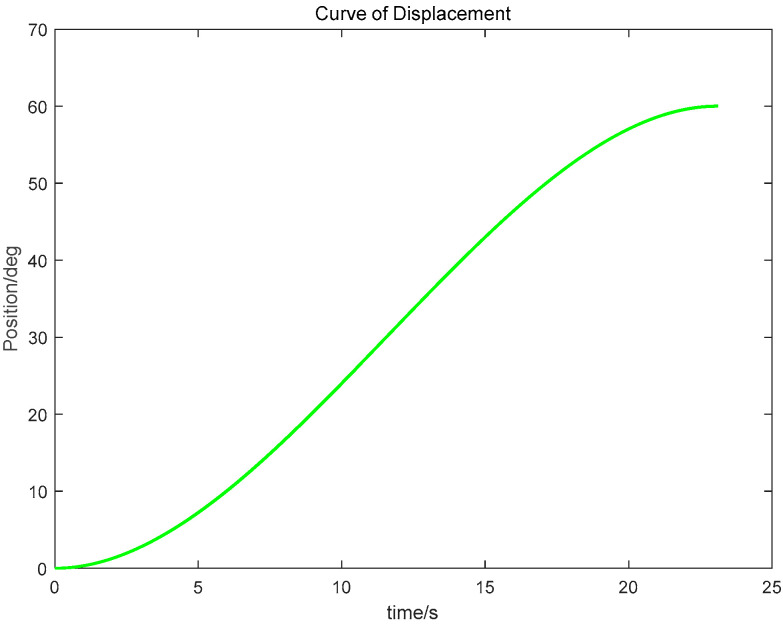
Displacement–time trajectory profile.

**Figure 7:**
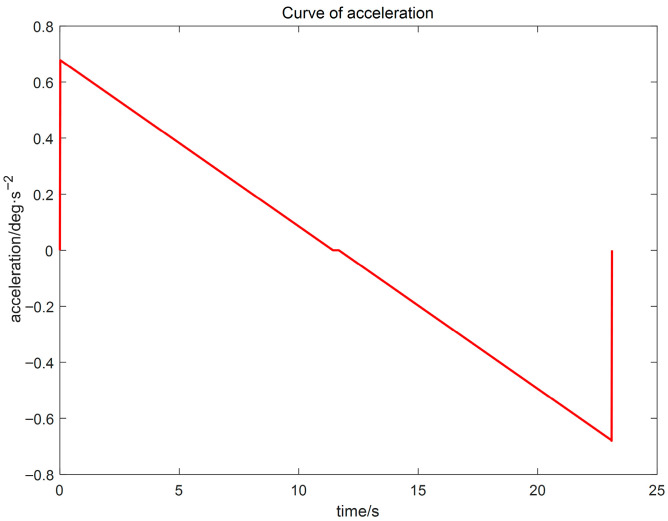
Acceleration–time profile.

(2) Establish the relationship between t_1_, t_2_, and total time t_3_, and use the central difference method to derive the acceleration of the forearm. Construct an energy cost function. By combining this function with the displacement equation and assigning weight values, a composite multi-objective function is obtained. Through global search by the exploration group and local optimization by the utilization group, the current optimal solution is determined. If the maximum preset number of iterations is reached, the iteration stops.

(3) Using the proposed GGO-based optimization under the prescribed constraints, the optimal switching times t_1_ and t_2_ were determined. The resulting kinematic profiles (velocity, displacement, acceleration, and jerk versus time) were then generated in MATLAB for subsequent analysis, as presented in Figure 5, Figure 6 and Figure 7.

The simulation results indicate that the proposed polynomial interpolation–based trajectory planning method enables the manipulator’s angular velocity to vary continuously and smoothly throughout the entire motion. This significantly mitigates mechanical vibration and impact induced by abrupt velocity variations, thereby helping to reduce oscillations of the end-effector. Moreover, the method imposes a lower computational burden on the control system, and the acceleration variation can be interpreted more intuitively substituting the optimal solution *t*_3_ into the energy cost function, the optimal vibration energy index under the prescribed weight constraint can be obtained. The double-parabola–trapezoidal velocity planning profile derived from the Graylag Goose Optimization (GGO) parameters is shown in Figure 5. Overall, the profile is relatively smooth, with no abrupt changes in velocity. Moreover, as indicated by the displacement curve in Figure 6 and the acceleration curve in Figure 7, the angular variation is gradual, and the acceleration exhibits no pronounced fluctuations.

## 5. Comparison Under Different Initial Input Conditions

**Figure 8:**
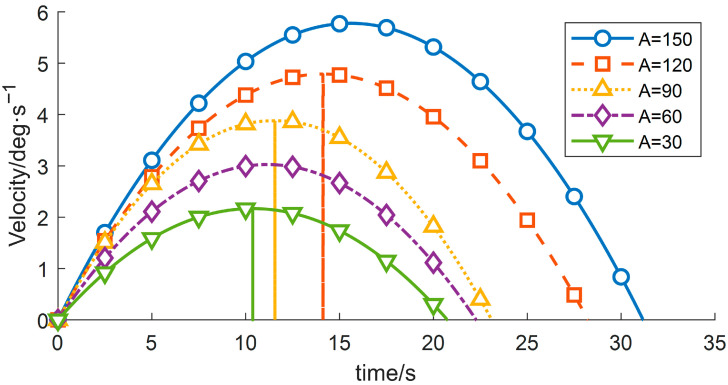
Velocity-time profiles for different planned displacements.

**Figure 9:**
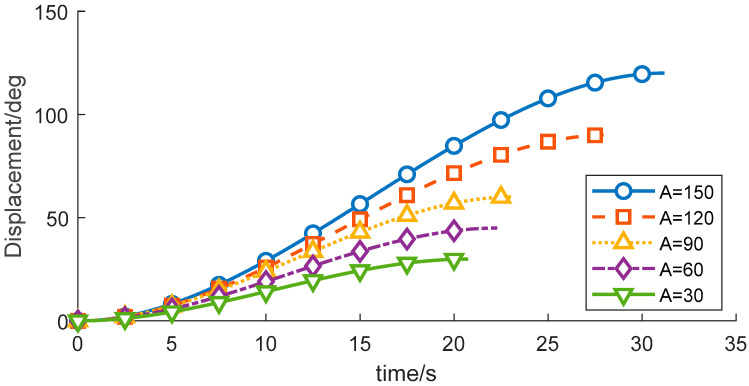
Displacement-time profiles for different planned displacements.

**Figure 10:**
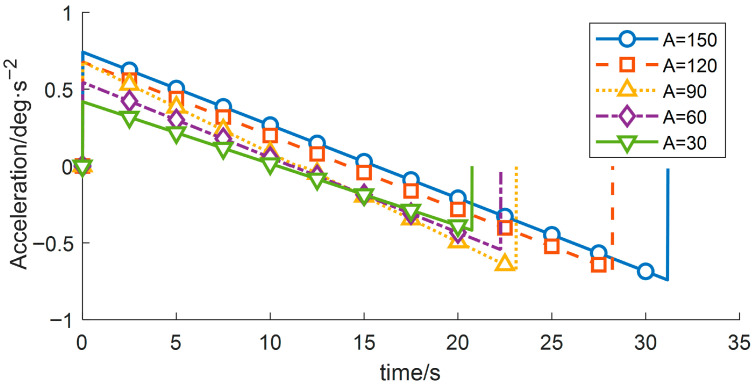
Acceleration-time profiles for different planned displacements.

(1) By modifying the motion range of the wafer-transfer manipulator, i.e., varying the angular displacement A, the corresponding trajectories and the residual vibration energy can be rapidly fitted through iterative computation. With all other conditions unchanged, the experimental results are summarized in Table 5 and Figure 8, Figure 9 and Figure 10.

As indicated in Table 5, the residual vibration energy is strongly influenced by the total angular displacement. When the angular change increases from 60° to 90°, the residual vibration energy rises by 103.28%. Therefore, constraining the forearm angular variation to within 60° can significantly suppress vibrations induced by the flexible joint.

**Figure 11:**
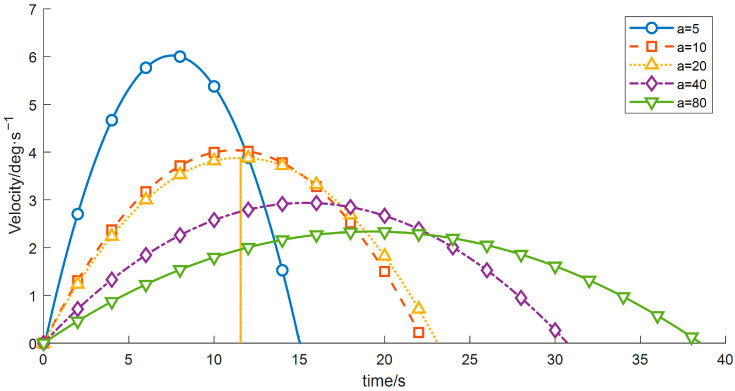
Velocity-time profiles for different start acceleration.

**Figure 12:**
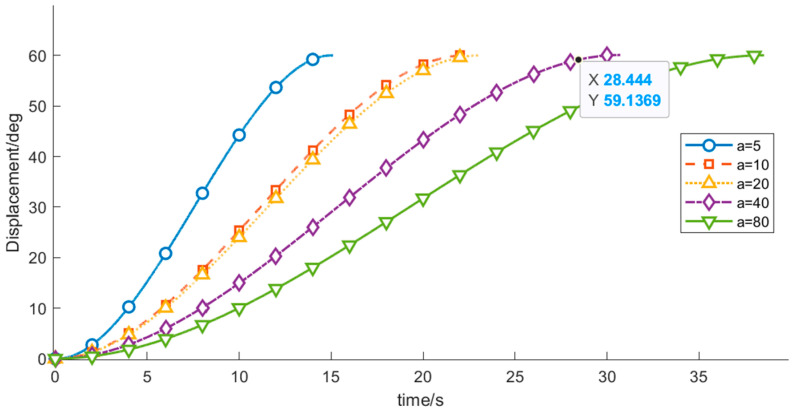
Displacement–time profiles for different start acceleration.

**Figure 13:**
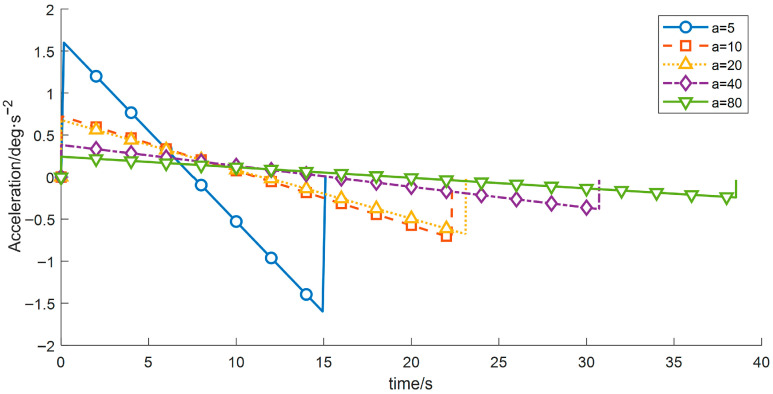
Acceleration–time profiles for different start acceleration.

(2) To investigate the influence of the start-up acceleration on the motion performance and vibration characteristics of the wafer-transfer manipulator, comparative experiments were conducted by varying the start-up acceleration *a* while keeping the other control parameters constant. The results are listed in Table 6 and Figure 11, Figure 12 and Figure 13.

As shown in Table 6, as *a* increases from 5 to 80, the overall operation time exhibits a decreasing trend, whereas the vibration energy index increases rapidly in a nonlinear manner. These results demonstrate that a larger start-up acceleration is beneficial for reducing the operation time and improving transfer efficiency. However, excessive start-up acceleration introduces stronger impact loads and excites high-frequency modes of the manipulator, leading to a substantial increase in vibration. Hence, a typical trade-off exists between efficiency and vibration suppression. Considering both efficiency and stability, a start-up acceleration of approximately *a* ≈ 20 deg/s^2^ can be regarded as a compromise range that balances operation time and vibration mitigation, and this range is advantageous for subsequent optimization and analysis.

**Figure 14:**
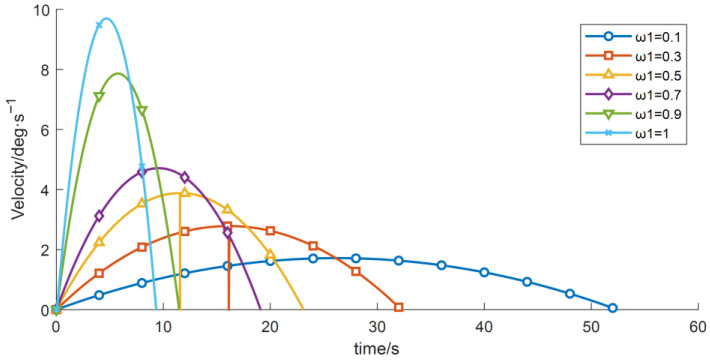
Velocity-time profiles for different time weight.

**Figure 15:**
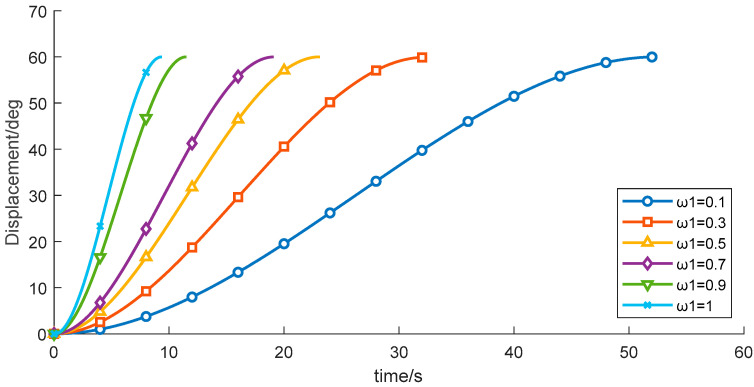
Displacement–time profiles for different start acceleration time weight.

**Figure 16:**
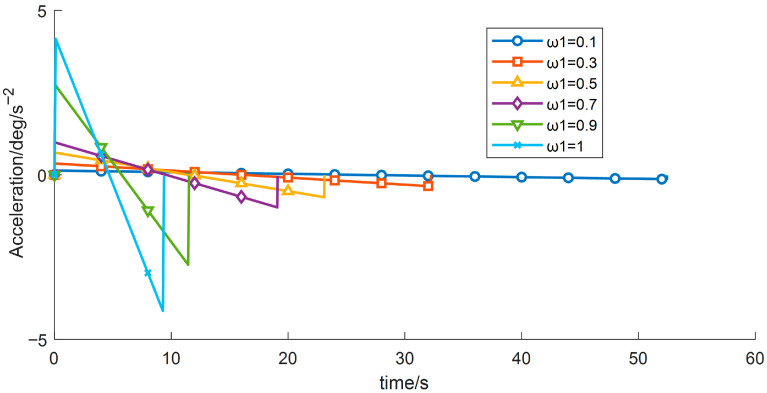
Acceleration–time profiles for different time weight.

(3) With the other control parameters unchanged, comparative experiments were performed by varying the time-weighting factor w1, the results are shown in Table 7 and Figure 14, Figure 15 and Figure 16. The experimental data indicate that, under the given constraints and operating conditions, increasing w1 gradually reduces the total time obtained from the objective function. When w1 > 0.9, the trajectory duration decreases markedly, while the vibration energy index increases sharply.

Table 7 shows that the sensitivity of both the total operation time and the vibration energy index to w1 is relatively low, and the current optimization results therefore lie in an approximately flat Pareto region. Overall, although increasing the time weight reduces the operation time, it may induce higher vibration energy, implying greater instability and/or energy consumption.

## 6. Simulation Analysis Based on Simulink

**Figure 17:**
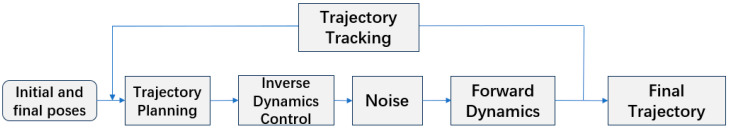
Robotic Arm Simulation System Flowchart.

In this study, a Simulink-based trajectory simulation platform was Established. The optimal trajectory parameters obtained from the optimization algorithm were imported into the platform to simulate and evaluate the manipulator’s stability under external disturbances. The external disturbance shown in Figure 17 is modeled as a bounded random torque applied to the joint actuator to emulate unmodeled dynamics and environmental perturbations in wafer-handling operations. The disturbance is defined as:

(20) $$\tau_{d}(t)=\tau_{\max}\xi (t),\;\;\xi (t)\in [-0.1,\;0.1]$$

where *ξ*(t) is a zero-mean bounded random process and *τ*_max_ denotes the disturbance bound. In the Simulink implementation, ξ(t) is generated using a random source (e.g., a band-limited noise or uniform random sequence) and then constrained by saturation to ensure |*τ_d_*(t)| ≤ *τ*_max_. This bounded setting provides a physically meaningful robustness test while avoiding unrealistically large excitation.

This represents unmodeled dynamics and external perturbations typically encountered in industrial wafer-handling environments.

**Figure 18:**
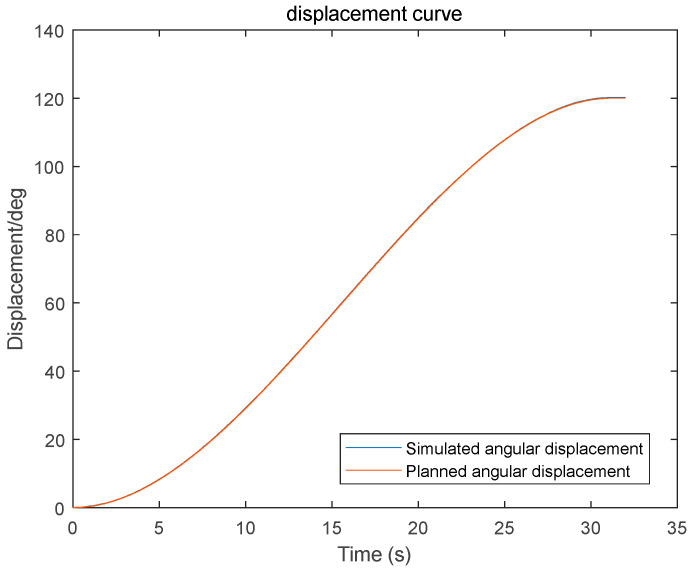
Robotic Arm Displacement Curve.

**Figure 19:**
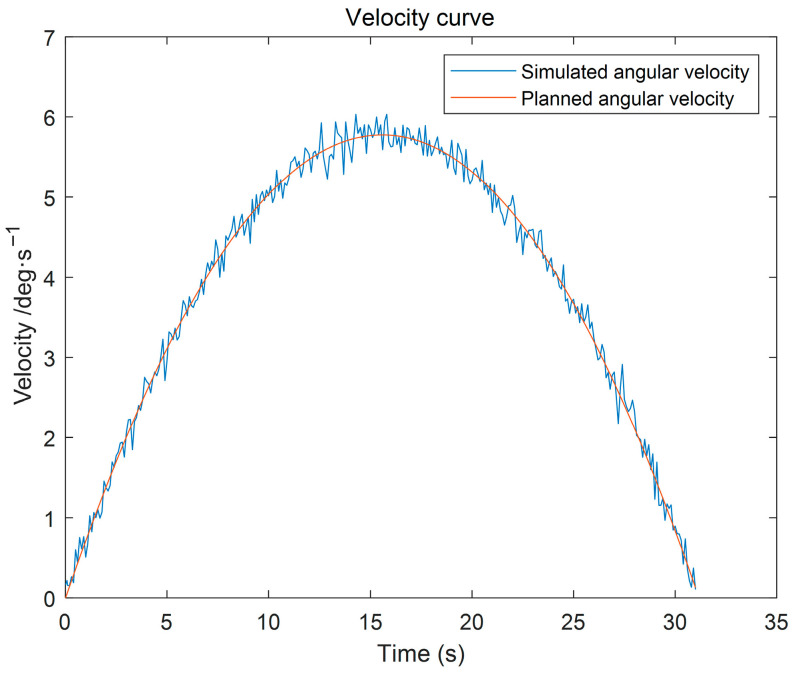
Robotic Arm Velocity Curve.

**Figure 20:**
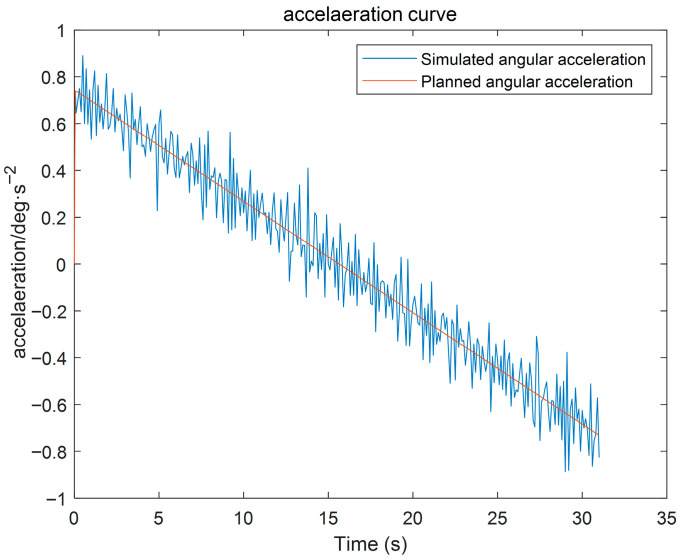
Robotic Arm Acceleration Curve.

To control the robot motion, the Simulink control program first initializes the system by setting the manipulator coordinates to the prescribed initial state [39]. Based on the planned trajectory parameters obtained from the preceding simulations, a smooth-function output module is constructed to generate the reference profiles of the joint angle, angular velocity, and angular acceleration. Meanwhile, a random disturbance module is connected externally to provide an excitation torque, which is applied as the external input torque *τ* to the dynamic planning module. A closed-loop control function is then established according to the governing dynamic equations. The output angular acceleration is integrated twice to obtain the simulated angular velocity and angular displacement, and the resulting data are exported to spreadsheets. Furthermore, inverse kinematics is employed to inversely infer the effect of the external disturbance and back-calculate the corresponding driving torque, thereby completing the closed-loop simulation test module [40]. In this study, angular displacement, angular velocity, angular acceleration, and joint torque are adopted as evaluation metrics to assess the time-planning performance and vibration-suppression capability of the wafer-transfer robot using the Simulink test platform. The integrated simulation platform is executed, and the Scope outputs are exported to the workspace via the To Workspace function, yielding the simulation results shown in Figure 18, Figure 19 and Figure 20.

As indicated by the simulation results in Figure 18, the planned joint angular displacement profile of the forearm joint is in close agreement with the simulated tracking angular displacement over the entire motion interval. The two curves almost overlap in the initial stage, the acceleration and constant-velocity transition stage, and the final deceleration stage, exhibiting an overall smooth S-shaped trajectory. In terms of tracking performance, the tracking angular displacement shows no evident overshoot or oscillation throughout the motion, and the steady-state error at the end is virtually negligible, demonstrating that the system achieves favorable dynamic response characteristics and high steady-state accuracy.

As shown by the simulation results in Figure 19, the planned angular velocity of the forearm joint agrees well with the simulated tracking angular velocity over the entire motion interval, with only a negligible deviation observed in the vicinity of the peak velocity. No evident overshoot or oscillation is observed, indicating that the controller can accurately track the desired angular-velocity command and that the system exhibits high velocity-tracking accuracy and stability. Such a smooth velocity profile is beneficial for reducing vibration levels in the joint and links, and it also prevents inertial impacts induced by abrupt acceleration/deceleration, which is crucial for maintaining posture stability and operational safety during wafer handling.

Furthermore, the planned and tracked angular accelerations of the forearm joint remain highly consistent throughout the motion, as shown in Figure 19. Only minor local high-frequency fluctuations are present, and no abrupt spikes or severe reverse jumps are detected. Therefore, the proposed planning method can fundamentally suppress structural vibrations of the manipulator, thereby supporting the stable operation of the wafer-transfer robot.

Based on the simulation results shown in Figure 18, Figure 19 and Figure 20, the vibration suppression performance of the proposed trajectory planning method is further validated. Compared with conventional polynomial-based trajectory planning approaches, the planned joint angular displacement, velocity, and acceleration profiles exhibit smooth transitions without evident oscillations or abrupt fluctuations. In particular, the angular-displacement fluctuation is constrained within 0.01 deg/s, quantitatively indicating effective disturbance rejection and enhanced dynamic smoothness, thereby supporting the vibration-suppression effect of the proposed jerk-minimization trajectory planning strategy.

Moreover, by introducing the proposed improved multi-objective Gray Goose Optimization (MOGGO) algorithm, the solution quality of the dual-objective vibration suppression trajectory optimization problem is significantly enhanced, benefiting from improved population diversity and global search capability.

To further evaluate the robustness of the proposed method, random external torque disturbances are introduced into the system. Under such disturbances, the tracking responses remain highly consistent with the planned trajectories, and no significant overshoot or residual oscillation is observed during acceleration, constant-velocity, or deceleration phases. In particular, the angular velocity fluctuation is effectively constrained within a small range, indicating that high-frequency vibration components are significantly suppressed. These results confirm that minimizing the jerk-based vibration energy index leads to improved dynamic smoothness and enhanced vibration suppression capability of the wafer-handling robotic arm.

## 7. Conclusions

This paper addresses the micro-vibration problem of wafer transfer robotic arms and proposes and validates a vibration suppression trajectory optimization method based on the Graylag Goose Optimization (GGO) algorithm. After theoretical derivation and simulation tests, the following conclusions are drawn:

(1) This study established and analyzed the dynamic model of the wafer transfer robotic arm based on the Lagrange method, providing a theoretical foundation for vibration suppression trajectory planning. The model fully considers the flexible dynamic characteristics of the robotic arm and accurately describes the system’s dynamic behavior.

(2) By combining a multi-segment polynomial trajectory planning method with a residual vibration energy dissipation equation, a multi-objective optimization function targeting both time-optimal performance and vibration energy was constructed. By reasonably setting weight coefficients according to working conditions, a multi-objective coordinated optimization was achieved to ensure trajectory smoothness and minimal residual vibration energy.

(3) Simulation results show that when solving the dual-objective vibration suppression trajectory optimization, introducing the proposed improved multi-objective Gray Goose Optimization (MOGGO) algorithm effectively solves the dual-objective problem. The results indicate that the derived trajectory, when subjected to random external disturbances, maintains speed fluctuations basically within 0.01 deg/s, demonstrating good vibration suppression performance.

However, this study only performed Pareto optimization for two objectives and has not been extended to higher-dimensional, multi-objective optimization problems. Moreover, it is limited to SIMULINK and MATLAB simulation environments. Future work will expand to validation on a PLC platform and further explore the algorithm’s applicability under more complex operating conditions and with additional optimization objectives.

## Figures and Tables

**Table 1:** D-H Parameter Table of the Wafer Handling Robot Arm.

Arm/l*i*	$q_{i}$/°	$d_{i}$/mm	$l_{i}$/mm	$\alpha_{i}$/°
L_1_	0–120	0	240	0
L_2_	0–120	0	240	0

**Table 2:** Parameters of Components of the Wafer Transport Robotic Arm.

Structure	Value
Primary arm length/L_1_	240 mm
Secondary arm length/L_2_	240 mm
Mass of Primary arm/m_1_	2.757 kg
Mass of secondary arm/m_2_	2.165 kg
The center of gravity of the primary arm/Lc_1_	0.4 L_1_
The center of gravity of the secondary arm/Lc1	0.5 L_2_

**Table 3:** Origin Parameter of graylag goose population.

Parameter	Value
Population size/N	50
Maximum number of iterations/M	200
Minimum time interval/δ	0.1

**Table 4:** CEC Function Test Results.

Function		PSO	NSGAII	GWO	WOA	DBO	MOGGO
F4	Mean	1.83 × 10^8^	9.87 × 10^6^	1.30 × 10^7^	4.23 × 10^7^	3.02 × 10^8^	6.62 × 10^4^
	Std	6.78 × 10^8^	9.83 × 10^6^	7.06 × 10^7^	9.44 × 10^7^	2.10 × 10^8^	3.91 × 10^4^
F5	Mean	3.78 × 10^7^	1.62 × 10^6^	3.37 × 10^6^	7.36 × 10^5^	1.06 × 10^5^	8.5 × 10^3^
	Std	5.25 × 10^7^	7.19 × 10^6^	4.56 × 10^6^	6.15 × 10^6^	2.12 × 10^6^	9.47 × 10^3^
F9	Mean	−557.91	−557.61	−556.16	−556.8	−557.27	−558.48
	Std	2.6599	1.8035	2.2561	1.8668	1.8109	3.6466
F14	Mean	1.97 × 10^4^	1.11 × 10^3^	9.82 × 10^3^	9.93 × 10^3^	7.41 × 10^3^	7.95 × 10^3^
	Std	1.25 × 10^4^	4.59 × 10^3^	3.28 × 10^3^	3.16 × 10^3^	4.12 × 10^3^	871.7893
F15	Mean	2.05 × 10^4^	1.24 × 10^4^	9.4 × 10^3^	8.76 × 10^3^	9.21 × 10^3^	7.35 × 10^3^
	Std	1.13 × 10^4^	4.50 × 10^3^	2.52 × 10^3^	3.12 × 10^3^	2.26 × 10^3^	1.08 × 10^3^
F16	Mean	204.0242	203.6665	203.8356	204.1670	203.9555	203.9054
	Std	0.6995	0.3577	0.3197	0.3353	0.4455	1.3284
F21	Mean	7.54 × 10^5^	1.38 × 10^5^	4.06 × 10^4^	5.70 × 10^5^	8.94 × 10^4^	2.98 × 10^3^
	Std	7.43 × 10^5^	3.44 × 10^5^	2.18 × 10^5^	2.52 × 10^5^	2.76 × 10^5^	249.0022
F23	Mean	2.14 × 10^4^	1.24 × 10^4^	1.09 × 10^4^	1.09 × 10^4^	1.09 × 10^4^	9.63 × 10^3^
	Std	1.26 × 10^4^	4.13 × 10^3^	2.91 × 10^3^	2.37 × 10^3^	1.96 × 10^3^	819.0361

**Table 5:** Variation in Objective Function Values of Wafer Transfer Robot Arm at Different Displacements.

Displacement	Runtime	Vibration Energy Index E_vib_
150	16.8	347.8261
120	13.8	347.8261
90	9.633	339.394
60	7.083	166.957
30	6.033	164.882

**Table 6:** Variation in Objective Function Values of Wafer Transfer Robot Arm at Different Startup Acceleration.

Startup Acceleration	Runtime	Vibration Energy Index E_vib_
−5	12.6952	32.8125
10	13.9619	131.2500
20	8.2476	525.0000
40	5.6347	2149.8891
80	2.0144	9029.1039

**Table 7:** Variation in Objective Function Values of Wafer Transfer Robot Arm at Different Time Weight.

Time Weight	Runtime	Vibration Energy Index E_vib_
0.1	22.0667	165.5172
0.3	22.0666	165.5172
0.5	21.4938	170.589
0.7	19.583	190.022
0.9	16.1414	239.1657
1.0	4.1635	69,522.553

## Data Availability

The original contributions presented in this study are included in the article. Further inquiries can be directed to the corresponding author.
